# Supplementary material for: Screening Vitis Genotypes for Responses to Botrytis cinerea and Evaluation of Antioxidant Enzymes, Reactive Oxygen Species and Jasmonic Acid in Resistant and Susceptible Hosts
Source: Molecules. 2018 Dec 20;24(1):5. doi: 10.3390/molecules24010005 (PMC6337682; doi:10.3390/molecules24010005)
Supplement: Supplementary file 1 [file molecules-24-00005-s001.pdf]

Screening of *Vitis* genotypes against *Botrytis cinerea* and response of antioxidant enzymes, reactive oxygen species and jasmonic acid in resistant and susceptible-hosts

Mati Ur Rahman [1, 2†]. MuhammadHanif [1, 2†]. Ran Wan [1, 3]. Xiaoqing Hou [1, 2]. Bilal Ahmad [1, 2]. Xiping Wang [1, 2*]

[1]State Key Laboratory of Crop Stress Biology in Arid Areas, College of Horticulture, Northwest A&F University, Yangling, Shaanxi 712100, China

[2]Key Laboratory of Horticultural Plant Biology and Germplasm Innovation in Northwest China, Ministry of Agriculture, Northwest A&F University, Yangling, Shaanxi 712100, China

[3] College of Horticulture, Henan Agriculture University, Zhengzhou, Henan 450002, China

[†] These authors have contributed equally to this research work.

*Corresponding author: wangxiping@nwsuaf.edu.cn

Table S1. *Vitis* species evaluated in this study are listed with their botanical names, author names and plant family in the table given below.

**S.No.**	**Species**	**Family name**	**Authors name**
1.	*Vitis vinifera* L.	*Vitaceae*	Carl Linnaeus
2.	*Vitis labrusca* L.	*Vitaceae*	Carl Linnaeus
3.	*Vitis amurensis* Rupr.	*Vitaceae*	Franz Josef Ruprecht
4.	*Vitis davidii* Foex	*Vitaceae*	Gustave Foëx
5.	*Vitis riparia* Michx.	*Vitaceae*	André Michaux

Table S2 .Lesions percentage on the leaves of 81 *Vitis* genotypes infected with *B. cinerea* from 2016 to 2017.

Specises	**Name of cultivars**	**Lesions percentages (%)**	
		**2016**	**2017**
Vitis vinifera L	Beauty Seedless	69.6 ± 1.53	71.0 ± 2.00
V. vinefera L x V.Amurensis Rupr	Beibinghong	74.2 ± 0.93	75.1 ± 0.81
V. vinefera L x V.Amurensis Rupr	Beichun	90.6 ± 1.15	91.3 ± 1.53
V. vinefera L x V.Amurensis Rupr	Beihong	23.5 ± 0.62	22.6 ± 0.61
Vitis vinifera L	Bixiang Seedless	82.0 ± 1.73	81.9 ± 1.82
Vitis vinifera L	Black Rose	69.0 ± 2.00	67.0 ± 2.60
Vitis vinifera L	Blue French	70.0 ± 1.00	69.7 ± 2.10
Vitis vinifera L	Blush Seedless	54.3 ± 1.15	56.6 ± 1.53
Vitis vinifera L	Cabernet Sauvignon	9.3 ± 0.58	12.0 ± 0.00
Vitis vinifera L	Canner Seedless	20.5 ± 0.87	20.6 ± 0.76
Vitis vinifera L	Cannero	70.5 ± 0.87	71.5 ± 0.50
Vitis vinifera L	Carignan	96.5 ± 1.32	97.5 ± 0.50
Vitis vinifera L	Chardonnay	92.0 ± 1.73	90.0 ± 1.73
Vitis vinifera L	Chenin Blanc	18.0 ± 1.00	20.0 ± 1.7
Vitis vinifera L	Cinsault	90.0 ± 2.00	87.3± 1.50
Vitis vinifera L	Crimson seedless	95.3 ± 2.08	96.4 ± 1.69
Vitis vinifera L	Dabai Grape	66.3 ± 1.53	64.7 ± 1.50
V.vineferaxV.lbrusca L.	Dongfangzhixing	92.3 ± 1.53	90.3 ± 1.53
Vitis vinifera L	Early Muscat	94.0 ± 1.00	96.0 ± 2.00
Vitis vinifera L	Flame Seedless	25.3 ± 1.53	27.7 ± 1.50
Vitis vinifera L	Fresno Seedless	25.2± 2.42	22.6 ± 2.80

V.vineferaxV.lbrusca L.	Gold Finger	73.3 ± 2.89	75.6 ± 2.08
V.vineferaxV.lbrusca L.	Heibaladuo	22.6 ± 0.58	24.0 ± 0.95
Vitis vinifera L	Heisetiancai	94.9 ± 0.85	93.3 ± 0.61
Vitis vinifera L	Hongmunage	44.1 ± 0.76	43.8 ± 0.76
V.vineferaxV.Lbrusca L.	Hu tai 8	94.2 ± 0.64	94.1 ± 0.76
Vitis vinifera L	Italian Riesling	44.6 ± 0.58	42.7 ± 0.60
Vitis vinifera L	Jing xiu	85.0 ± 0.40	84.1 ± 0.61
Vitis vinifera L	Jingkejing	69.6 ± 1.15	73.6 ± 1.53
Vitis vinifera L	Jingzaojing	83.6 ± 1.53	84.3 ± 1.53
Vitis vinifera L	Jinxiangyu	66.3 ± 1.53	68.0 ± 1.00
Vitis vinifera L	Jumeigui	8.9 ± 0.15	8.7 ± 0.52
V. riparia. × V. labrusca L.	Kangsan	15.7 ± 0.70	16.0 ± 0.30
V.vineferaxV.lbrusca L.	Kyoho	58.2 ± 0.68	58.2 ± 0.64
Vitis vinifera L	Lady Finger	71.8 ± 2.02	74.5 ± 1.32
Vitis vinefera	Long Emprer	33.3 ± 2.08	32.0 ± 2.65
Vitis vinifera L	Malta Seedless	60.6 ± 2.08	57.0 ± 1.00
Vitis vinifera L	Manaizi	92.6 ± 0.58	93.3 ± 0.58
Vitis vinifera L	Marselan	15.6 ± 1.53	16.3 ± 0.58
Vitis vinifera L	May Purple	25.6 ± 5.13	23.7 ± 4.70
Vitis vinifera L	Merlot	82.8 ± 1.26	86.5 ± 1.32
Vitis vinifera L	Moldova	91.3 ± 0.70	94.3 ± 0.58
Vitis vinifera L	Mr Mori	98.1 ± 0.76	91.8 ± 0.29
Vitis vinifera L	Muscat Blanc	46.8 ± 1.76	48.6 ± 1.53
Vitis vinifera L	Muscat of Alexandia	23.3 ± 2.08	24.3 ± 1.20
Vitis vinifera L	Muscat Rose	62.6 ± 0.58	66.3 ± 0.60

V.vineferaxV.lbrusca L.	Neptune Seedless	28.1 ± 0.76	26.5 ± 0.87
Vitis vinifera L	Otilia Seedless	87.0 ± 2.65	85.6 ± 2.08
Vitis vinifera L	Pearl of Csaba	75.6 ± 0.58	73.7 ± 0.60
Vitis vinifera L	Perlette	45.8 ± 0.76	46.6 ± 0.58
Vitis vinifera L	Phoenix NO.51	80.6 ± 1.15	82.0 ± 1.00
Vitis vinifera L	Pinot Noir	89.3 ± 0.58	86.7± 0.60
Vitis vinifera L	Prince Seedless	9.3 ± 1.15	9.6 ± 0.58
Vitis vinifera L	Qiumanai	22.3 ± 1.53	23.3 ± 1.50
Vitis vinifera L	Queen	10.6 ± 1.15	10.7 ± 1.50
Vitis vinifera L	Red Globe	73.2 ± 0.72	71.5 ± 0.51
Vitis vinifera L	Red Hanepoot	71.3 ± 2.08	73.0 ± 2.60
Vitis vinifera L	Riesling	98.1 ± 0.76	99.0 ± 1.00
Vitis vinifera L	Rizamat	19.3 ± 0.58	20.1 ± 0.76
Vitis vinifera L	Rkatsiteli	61.6 ± 2.52	68.3 ± 2.10
Vitis vinifera L	Ruby Seedless	77.3 ± 2.08	78.3 ± 2.89
Vitis vinifera L	Russian Seedless	16.6 ± 1.53	17.6 ± 1.15
Vitis vinifera L	Sangiovese	62.9 ± 0.10	63.2 ± 0.68
Vitis vinifera L	Sauvignon Blanc	55.0 ± 2.65	56.3 ± 2.10
Vitis vinifera L	Semillon	88.0 ± 0.00	82.3 ± 0.58
Vitis vinifera L	Sultanina Rose	38.6 ± 1.15	38.3 ± 0.60
V.vineferaxV.lbrusca L.	Summer Black	93.7 ± 0.86	94.1 ± 0.72
Vitis vinifera L	Thompson Seedless	63.3 ± 1.23	63.0 ± 1.10
V.vineferaxV.lbrusca L.	Tian yuan qi	87.8 ± 1.04	86.2 ± 0.72
Vitis vinifera L	Tokay	90.3 ± 1.15	91.3 ± 1.53
Vitis vinifera L	Ugni Blanc	92.6 ± 3.51	96.6 ± 2.31

Vitis vinifera L	Victoria	84.0 ± 2.00	83.3 ± 2.08
Vitis vinifera L	Wink	93.2 ± 0.64	94.7 ± 0.49
Vitis amurensis	Xuelanhong	91.5 ± 1.80	92.0 ± 1.01
Vitis vinifera L	Yangle	34.3 ± 2.52	36.0 ± 2.65
Vitis vinifera L	Yatomi Rosa	88.8 ± 1.03	90.7 ± 1.47
Vitis vinifera L	Yilixiang	57.6 ± 1.53	56.0 ± 2.65
V.vineferaxV.lbrusca L.	Zaojinxiang	11.3 ± 1.15	10.3 ± 1.53
Vitis vinifera L	Zhana	46.3 ± 1.53	47.7 ± 0.60
Vitis davidii	Ziqiu	2.7 ± 0.44	3.0 ± 0.80
V.vineferaxV.lbrusca L.	Zuijinxiang	53.1 ± 1.04	51.6 ± 1.15
